# The Molecular Basis of Drug Resistance against Hepatitis C Virus NS3/4A Protease Inhibitors

**DOI:** 10.1371/journal.ppat.1002832

**Published:** 2012-07-26

**Authors:** Keith P. Romano, Akbar Ali, Cihan Aydin, Djade Soumana, Ayşegül Özen, Laura M. Deveau, Casey Silver, Hong Cao, Alicia Newton, Christos J. Petropoulos, Wei Huang, Celia A. Schiffer

**Affiliations:** 1 Department of Biochemistry and Molecular Pharmacology, University of Massachusetts Medical School, Worcester, Massachusetts, United States of America; 2 Monogram Biosciences, San Francisco, California, United States of America; Fundación Instituto Leloir-CONICET, Argentina

## Abstract

Hepatitis C virus (HCV) infects over 170 million people worldwide and is the leading cause of chronic liver diseases, including cirrhosis, liver failure, and liver cancer. Available antiviral therapies cause severe side effects and are effective only for a subset of patients, though treatment outcomes have recently been improved by the combination therapy now including boceprevir and telaprevir, which inhibit the viral NS3/4A protease. Despite extensive efforts to develop more potent next-generation protease inhibitors, however, the long-term efficacy of this drug class is challenged by the rapid emergence of resistance. Single-site mutations at protease residues R155, A156 and D168 confer resistance to nearly all inhibitors in clinical development. Thus, developing the next-generation of drugs that retain activity against a broader spectrum of resistant viral variants requires a comprehensive understanding of the molecular basis of drug resistance. In this study, 16 high-resolution crystal structures of four representative protease inhibitors – telaprevir, danoprevir, vaniprevir and MK-5172 – in complex with the wild-type protease and three major drug-resistant variants R155K, A156T and D168A, reveal unique molecular underpinnings of resistance to each drug. The drugs exhibit differential susceptibilities to these protease variants in both enzymatic and antiviral assays. Telaprevir, danoprevir and vaniprevir interact directly with sites that confer resistance upon mutation, while MK-5172 interacts in a unique conformation with the catalytic triad. This novel mode of MK-5172 binding explains its retained potency against two multi-drug-resistant variants, R155K and D168A. These findings define the molecular basis of HCV N3/4A protease inhibitor resistance and provide potential strategies for designing robust therapies against this rapidly evolving virus.

## Author Summary

Hepatitis C virus (HCV) infects over 170 million people worldwide and is the leading cause of chronic liver diseases, including cirrhosis, liver failure, and liver cancer. New classes of directly-acting antiviral agents that target various HCV enzymes are being developed. Two such drugs that target the essential HCV NS3/4A protease are approved by the FDA and several others are at various stages of clinical development. These drugs, when used in combination with pegylated interferon and ribavirin, significantly improve treatment outcomes. However HCV evolves very quickly and drug resistance develops against directly-acting antiviral agents. Thus, despite the therapeutic success of NS3/4A protease inhibitors, their long-term effectiveness is challenged by drug resistance. Our study explains in atomic detail how and why drug resistance occurs for four chemically representative protease inhibitors –telaprevir, danoprevir, vaniprevir and MK-5172. Potentially with this knowledge, new drugs could be developed that are less susceptible to drug resistance. More generally, understanding the underlying mechanisms by which drug resistance occurs can be incorporated in drug development to many quickly evolving diseases.

## Introduction

Hepatitis C virus (HCV) is a genetically diverse positive-stranded RNA virus of the *Flaviviridae* family infecting an estimated 170 million people worldwide [1], [2]. Based on genetic diversity, HCV is divided into six major genotypes (genotypes 1–6) and numerous subtypes with different geographic distributions; genotypes 1 and 3 are the most prevalent worldwide [3]. HCV infection is the leading cause of chronic liver disease that persists for decades and eventually progresses to cirrhosis, liver failure, or liver cancer [4]. The current anti-HCV standard of care is a combination of pegylated interferon (Peg-IFN), ribavirin (RBV), and boceprevir or telaprevir, two recently approved antiviral agents targeting the viral NS3/4A protease [5]. Sustained virologic response (SVR) –which is tantamount to cure–is achieved only in a subset of treated patients, depending on a combination of viral and host-cell genetic factors [6]–[10]. For example, a human polymorphism at the IL28B gene is associated with poor interferon response [11]. Most patients undergoing interferon-based therapies also experience significant adverse effects, including flu-like symptoms, anemia, and depression [12]. Thus, current anti-HCV therapies are often not tolerated and ineffective for many patients, and novel direct-acting antiviral drugs are required for safer, more efficacious treatment.

**Figure 1:**
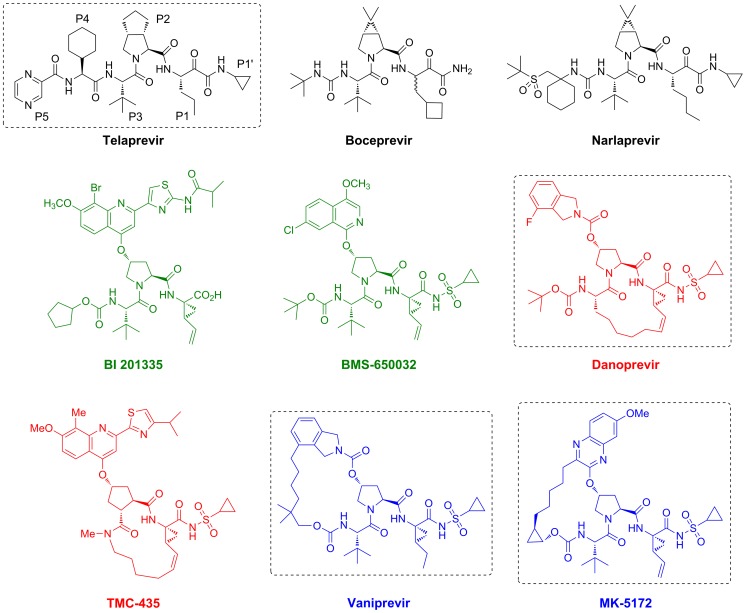
The chemical structures of NS3/4A protease inhibitors. The canonical nomenclature for drug moiety positioning is indicated using telaprevir. Telaprevir (black), danoprevir (red), vaniprevir and MK-5172 (blue) are representative of many other protease inhibitors in development. Telaprevir, recently approved for clinical use, is an acyclic ketoamide inhibitor that forms a reversible, covalent bond with the protease. Danoprevir, currently in phase II clinical trials, is a non-covalent acylsulfonamide inhibitor with a P1–P3 macrocycle. Vaniprevir and MK-5172 are also non-covalent acylsulfonamide inhibitors, but contain P2–P4 macrocycles. Vaniprevir and MK-5172 differ in the construction of their P2 moieties: vaniprevir contains a carbamate linkage between the P2 proline and the isoindoline moiety, whereas MK-5172 contains a shorter ether linkage between its P2 proline and the quinoxaline moiety.

Direct-acting antiviral agents have the potential to improve SVR rates and minimize treatment duration. The HCV NS3/4A protease – a chymotrypsin-like serine protease – is a prime therapeutic target that cleaves four known sites along the virally encoded polyprotein [13]. The NS3/4A protease also hydrolyzes two human proteins, TRIF and MAVS, which are part of the innate immune system, thereby confounding the innate immune response to viral infection [14], [15]. Pharmaceutical companies have invested significant effort in developing NS3/4A protease inhibitors. Proof-of-concept of antiviral efficacy was first demonstrated in 2002 with the macrocyclic inhibitor BILN-2061 (ciluprevir) [16], [17], which was later discontinued due to concerns about its cardiotoxicity [18]. As noted above, boceprevir [19] and telaprevir [20], [21] are two NS3/4A protease inhibitors recently approved by the Food and Drug Administration, marking an important milestone in anti-HCV research and drug development over the past two decades. Both boceprevir and telaprevir are linear ketoamide compounds that form a reversible, covalent bond with the catalytic serine of NS3/4A protease. Several non-covalent xprotease inhibitors have also advanced into human clinical trials; these inhibitors include both linear (BMS-650032 [22], BI 201335 [23]) and macrocyclic compounds, containing either a P1–P3 (danoprevir [24], TMC435 [25]) or a P2–P4 (vaniprevir [26], MK-5172 [27]) macrocycle (Figure 1).

The NS3/4A protease inhibitors rapidly reduce HCV RNA titers when administered as monotherapy [17], [28]–[31] and substantially improve SVR rates when given in combination with Peg-IFN and RBV [6]–[10], [32]–[34]. However, the high rate of HCV replication and poor fidelity of HCV's RNA-dependent RNA polymerase lead to heterogeneous virus populations in infected patients [35], [36]. These viral quasispecies exist at low levels in untreated patients, and resistant populations emerge under the selective pressure of direct-acting antiviral agents [36]–[38]. In the majority of patients undergoing protease inhibitor therapy, resistance develops rapidly due to overlapping but distinct sets of NS3/4A mutations [37]. In patients with genotype 1a, the R155K mutation causes resistance against nearly all inhibitors, but rarely occurs in genotype 1b patients [29], [30], [32], [37]–[42]. Instead, distinct resistance mutations arise in genotype 1b patients depending on the class of protease inhibitor used; A156 mutates in response to treatment with linear ketoamide protease inhibitors [39]–[41], while macrocyclic inhibitors more commonly select for D168A and R155K variants [29], [30], [32], [42]. Mutations at V36, T54, and V36+A155 are also associated with resistance to ketoamide inhibitors [39]–[41]. Variations in the patterns of resistant mutations arise from the complex interplay between genotype, replication rates, mutation rates, and the resulting effect of mutations on viral fitness and drug potency. Clearly, despite the benefits of combination therapy in improving SVR rates, the emergence of resistance challenges the long-term efficacy of NS3/4A protease inhibitors.

Most primary drug-resistance mutations in NS3/4A protease occur around the active site in regions where drugs protrude from the substrate binding space, defined as the substrate envelope, because these changes can preferentially disrupt drug binding with minimal effect on substrate binding and viral fitness [43]. The protease inhibitors danoprevir, TMC435, and boceprevir protrude from the substrate envelope in regions that correlate with known sites of resistance mutations. Notably, the large P2 moieties of danoprevir and TMC435 bind in the S2 subsite and extensively interact with residues R155, D168, and A156 [43], which mutate to confer multi-drug resistance [37], [38], [44]. These and other inhibitors with large P2 moieties derive much of their potency from binding in the S2 subsite [45], but how molecular changes at these residues selectively weaken inhibitor binding without compromising the binding of viral substrates is not clear. Elucidating the underlying molecular mechanisms of NS3/4A protease inhibitor resistance is therefore essential for developing new drugs that are less susceptible to resistance.

How single-site mutations at residues R155, A156 and D168 confer resistance against most protease inhibitors has not been elucidated in atomic detail. In this study, we report that four chemically representative protease inhibitors – telaprevir, danoprevir, vaniprevir and MK-5172 – exhibit distinct susceptibilities to the protease variants R155K, A156T and D168A (Table 1). Sixteen high-resolution crystal structures of inhibitors in complexwith the wild-type protease and three drug resistant variants reveal the molecular basis underlying the unique resistance profiles of these inhibitors (Table 2). The P2 quinoxaline moiety of MK-5172 stacks against the protease catalytic triad in a novel conformation, explaining its retained potency against R155K and D168A. The flexible P2 isoindoline moiety of danoprevir containing a P1–P3 macrocycle packs against the mutated surfaces of A156T and D168A variants, explaining its relatively higher activity against both protease variants. However, the isoindoline moiety in vaniprevir is constrained due to the P2–P4 macrocycle, resulting in significantly lower activity against all three variants. Thus, incorporating either quinoxaline or flexible substituents at the P2 proline confers clear advantages. Taken together, these data highlight potential strategies for designing novel drugs that retain potency against a broader spectrum of resistant viral variants.

**Table 1:** Drug susceptibilities against wild-type and resistant HCV clones and inhibitory activities against NS3/4A proteases.

	Replicon - IC_50_ (nM)^a^				Binding - *K*_i_ (nM)^a^			
Drug	WT	R155K	D168A	A156T	WT	R155K	D168A	A156T
Telaprevir (VX-950)	1030	5300 **(5.1)**	420 **(0.4)**	>50,000 **(>49)**	34.4±3.0	823±60 **(24)**	12.2±0.9 **(0.35)**	>10000 **(>291)**
Danoprevir (ITMN-191)	0.24	>100 **(>416)**	48 **(200)**	5.7 **(24)**	1.0±0.1	162±16 **(162)**	208±66 **(208)**	44.8±3.6 **(45)**
Vaniprevir (MK-7009)	0.34	>400 **(>1176)**	>400 **(>1176)**	176 **(518)**	0.74±0.07	554±64 **(749)**	2635±702 **(3561)**	958±162 **(1295)**
MK-5172	0.11	0.55 **(5)**	13 **(118)**	108 **(982)**	0.14±0.02	0.84±0.05 **(6)**	27.8±12.1 **(199)**	620±71 **(4429)**

a Numbers in parentheses reflect fold-change relative to wild-type; >indicates IC_50_ and *K*i values higher than the maximum drug concentration tested in the assay.

**Table 2:** X-ray data collection and crystallographic refinement statistics.

Drug	Telaprevir				Danoprevir				Vaniprevir				MK-5172			
Protease variant	WT	R155K	D168A	A156T	WT^i^	R155K^i^	D168A^i^	A156T^i^	WT^i^	R155K^i^	D168A^i^	A156T^i^	WT^t^	R155K^t^	D168A^t^	A156T^i^
PDB ID	3SV6	3SV7	3SV8	3SV9	3M5L	3SU0	3SU1	3SU2	3SU3	3SU4	3SU5	3SU6	3SUD	3SUE	3SUF	3SUG
Resolution (Å)	1.40	1.55	2.50	1.60	1.25	1.16	1.40	1.50	1.30	2.25	1.55	1.10	1.96	2.20	2.30	1.80
Space group	P2_1_2_1_2_1_	P2_1_2_1_2_1_	P4_1_2_1_2	P2_1_2_1_2_1_	P2_1_2_1_2_1_	P2_1_2_1_2_1_	P2_1_2_1_2_1_	P2_1_2_1_2_1_	P2_1_2_1_2_1_	P6_1_	P2_1_2_1_2_1_	P2_1_2_1_2_1_	P2_1_	P2_1_	P2_1_	P2_1_2_1_2_1_
Twin Law	-	-	-	-	-	-	-	-	-	-	-	-	-h, -k, h+l	-h, -k, h+l	-h, -k, h+l	-
Twin Fraction	-	-	-	-	-	-	-	-	-	-	-	-	0.42	0.42	0.43	-
Molecules in AU^b^	1	1	1	1	1	1	1	1	1	2	1	1	4	4	4	1
Cell dimensions:																
a (Å) =	55.1	55.3	69.5	54.8	55.2	55.3	55.0	54.9	55.1	85.8	55.1	55.0	56.1	56.3	56.0	53.9
b (Å) =	58.8	58.8	69.5	58.7	58.7	58.5	58.5	58.5	58.5	85.8	58.8	58.5	102.7	103.3	103.6	58.2
c (Å) =	60.3	60.4	79.1	60.7	61.1	60.6	60.0	60.0	60.3	97.4	60.0	59.8	73.3	73.5	73.5	62.0
β (°) =	90.0	90.0	90.0	90.0	90.0	90.0	90.0	90.0	90.0	90.0*	90.0	90.0	112.5	112.6	112.0	90.0
Completeness (%)	99.9	99.6	99.8	99.8	99.7	97.9	100.0	100.0	95.7	99.9	95.8	96.6	91.6	91.6	95.9	90.1
Measured reflections	39340	183407	91636	147126	317137	394396	233176	228689	196059	152278	190711	400120	101245	77906	107542	62460
Unique reflections	218617	29054	7137	26564	52645	67429	38857	31822	46251	19213	27508	76300	50198	36194	37671	16890
Average I/σ^a^	13.8	11.1	7.5	12.0	20.7	11.2	9.8	10.0	12.0	7.2	8.8	12.1	10.0	9.5	9.4	19.0
	(3.6)	(3.9)	(7.5)	(3.5)	(3.8)	(2.8)	(3.8)	(4.2)	(3.6)	(4.9)	(3.7)	(2.7)	(3.6)	(2.4)	(2.6)	(3.0)
Redundancy	5.6	6.3	12.8	5.5	6.0	5.8	6.0	7.2	4.2	7.9	6.9	5.2	2.0	2.2	2.9	3.7
R_sym_ (%)^a^ *^,^* c	3.5	6.1	11.0	4.5	4.6	4.1	4.3	4.3	4.1	7.7	6.9	4.4	6.0	6.7	6.3	2.8
	(41.2)	(37.6)	(58.8)	(40.0)	(31.5)	(39.9)	(45.2)	(45.3)	(29.6)	(37.9)	(44.0)	(28.3)	(19.5)	(36.2)	(38.7)	(30.9)
RMSD^d^ in:																
Bonds (Å) =	0.009	0.009	0.012	0.009	0.009	0.009	0.009	0.009	0.009	0.009	0.009	0.009	0.009	0.009	0.009	0.009
Angles (°) =	1.32	1.30	1.47	1.31	1.48	1.32	1.37	1.40	1.44	1.26	1.39	1.36	1.33	1.31	1.36	1.40
R_factor_ (%)^e^ =	15.8	16.5	20.5	17.2	15.0	15.3	15.7	15.4	16.4	16.9	16.5	15.0	18.3	18.5	19.7	19.4
R_free_ (%)^e^ =	17.1	19.9	28.3	19.7	16.8	17.2	17.8	17.7	18.2	22.3	18.3	16.5	23.8	22.7	25.6	23.0

i S139A protease mutant used for crystallization.

t pseudo-merohedral twin.

a values in parentheses are for the highest resolution shell.

b AU, asymmetric unit.

c R_sym_ = Σ|I−<I>|/ΣI, where I = observed intensity, <I> = average intensity over symmetry equivalent.

d RMSD, root mean square deviation.

e *R*_work_ = Σ∥*F_o_*|−|*F_c_*∥/Σ|*F_o_*|. *R*_free_ was calculated from 5% of reflections, chosen randomly, which were omitted from the refinement process.

*: γ = 120.0.

## Results

### Drug susceptibility assays

Drug activities were determined for telaprevir, danoprevir, vaniprevir and MK-5172 against wild-type genotype 1a HCV and resistant variants R155K, D168A, and A156T using viral replicon-based inhibition assays. The antiviral activities against the resistant variants trended with changes in binding affinities measured in enzyme inhibition assays (Table 1). Against wild-type protease, macrocyclic inhibitors danoprevir, vaniprevir and MK-5172 exhibited antiviral potencies in the sub nM range (IC_50_ = 0.24, 0.34 and 0.11 nM, respectively), while telaprevir potency was significantly lower (IC_50_ = 1030 nM), consistent with previous reports [46], [47]. Relative to the wild type, R155K caused large reductions in potency for danoprevir and vaniprevir, but MK-5172 remained highly active (R155K IC_50_ = 0.55 nM). Telaprevir potency was slightly better against D168A relative to the wild type, while danoprevir, vaniprevir and MK-5172 lost 100- to 1000-fold potency against D168A. However, both danoprevir and MK-5172 still were significantly more potent than telaprevir against D168A. Among the macrocyclic drugs, danoprevir and MK-5172 retained higher activities against D168A (D168A IC_50_ = 48 nM and 13 nM, respectively) relative to vaniprevir (D168A IC_50_>400 nM). Danoprevir also retained significantly higher potency against A156T (A156T IC_50_ = 5.7 nM), while the other three drugs incurred large-fold losses in potency. Notably, MK-5172, though active against the other two variants, lost significant potency against A156T (A156T IC_50_ = 108 nM). Thus, the four drugs exhibited varied susceptibilities to protease inhibitor-resistant viral variants R155K, D168A and A156T.

### Structure determination and analyses

To elucidate the underlying mechanism by which chemically diverse inhibitors bind to the wild-type protease and drug-resistant variants, crystal structures were determined for 16 inhibitor-protease complexes. These complexes include wild-type protease and resistant variants R155K, D168A and A156T each bound to telaprevir, danoprevir, vaniprevir and MK-5172, with resolutions ranging from 1.10–2.50 Å (Table 2); S139A protease variants were used except for telaprevir, which requires covalent bond formation with the serine 139 for efficient binding. These high-resolution data sets afforded very detailed structural interpretations of drug-protease binding.

**Figure 2:**
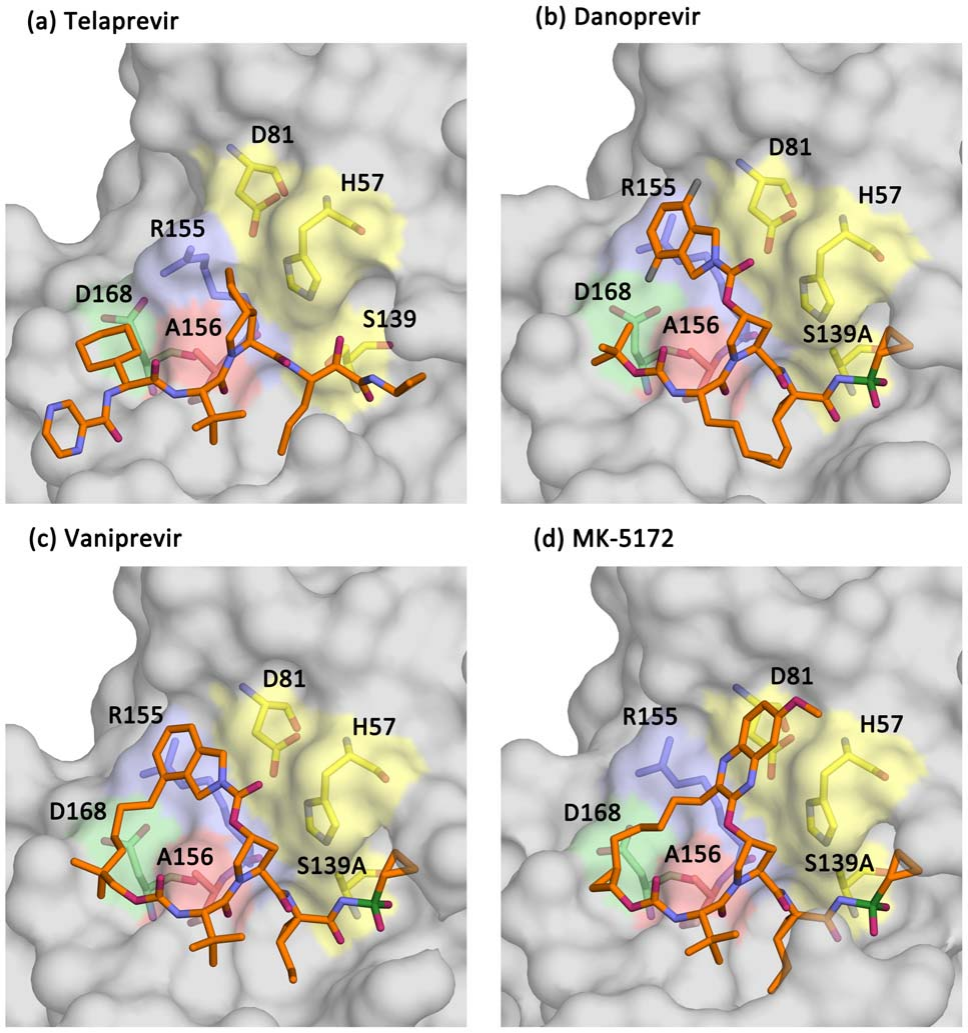
The binding conformations of telaprevir, danoprevir, vaniprevir and MK-5172. Surface representations of the wild-type protease in complex with (A) telaprevir, (B) danoprevir, (C) vaniprevir, and (D) MK-5172. The catalytic triad is shown in yellow and the R155, A156 and D168 side chains are highlighted in light-blue, pale-green and red, respectively.

**Figure 3:**
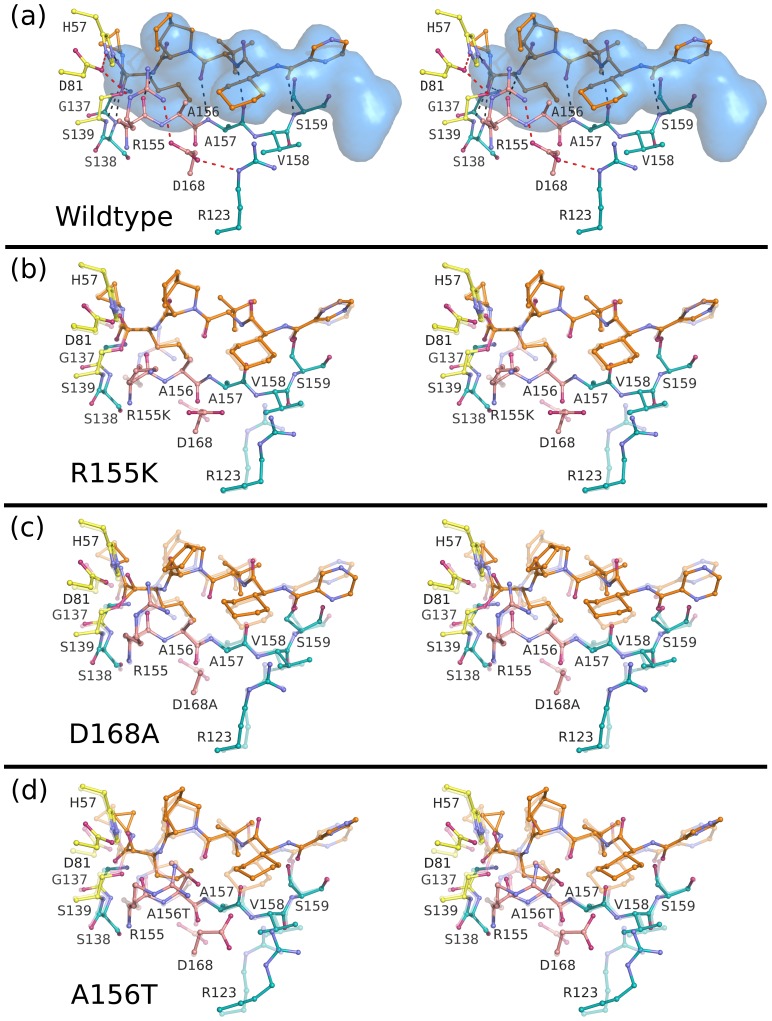
Stereo view of the telaprevir complexes. (A) Telaprevir bound to the wild-type protease with the substrate envelope in blue. Intra- and inter-molecular hydrogen bond interactions are marked as red and grey dashed lines. Telaprevir is also shown bound to the drug-resistant variants (B) R155K, (C) D168A and (D) A156T with the transparent coordinates representing the wild-type structure to better highlight the molecular changes of each mutation. In all cases, catalytic residues are depicted in yellow, the P2 subsite in pink, and the drug molecules in orange.

The binding conformations of telaprevir, danoprevir, vaniprevir and MK-5172 to the wild-type protease are shown in Figure 2 and Figure S1. In all complexes, inhibitors formed three common hydrogen bonds with the protease backbone (Table S1): (1) the P1 amide nitrogen with the carbonyl oxygen of R155, (2) the P3 carbonyl oxygen with the amide nitrogen of A157, and (3) the P3 amide nitrogen with the carbonyl oxygen of A157 (Figures 3A–[Fig ppat-1002832-g004] [Fig ppat-1002832-g005] 6A). The P5 amide nitrogen of telaprevir formed an additional hydrogen bond with the carbonyl oxygen of S159. In the telaprevir complex, the catalytic serine (S139) was covalently bound to the C-α carbon of the ketoamide warhead. The ketoamide oxygen sat in the oxyanion hole and interacted with the backbone amide nitrogens of protease residues 137–139, while the Nε nitrogen of H57 hydrogen bonded with the keto oxygen. The acylsulfonamide groups of danoprevir, vaniprevir and MK-5172 were also positioned in the oxyanion hole, hydrogen bonding with the same set of backbone amide nitrogens, as observed previously for the TMC435 and danoprevir structures [43], [45]. Meanwhile the Nε nitrogen of H57 interacted with the sulfonamide nitrogen in these complexes, suggesting that the Nε atoms were deprotonated. Thus, many of these classes of inhibitors overlap in several key interactions with the protease.

**Figure 4 ppat-1002832-g004:**
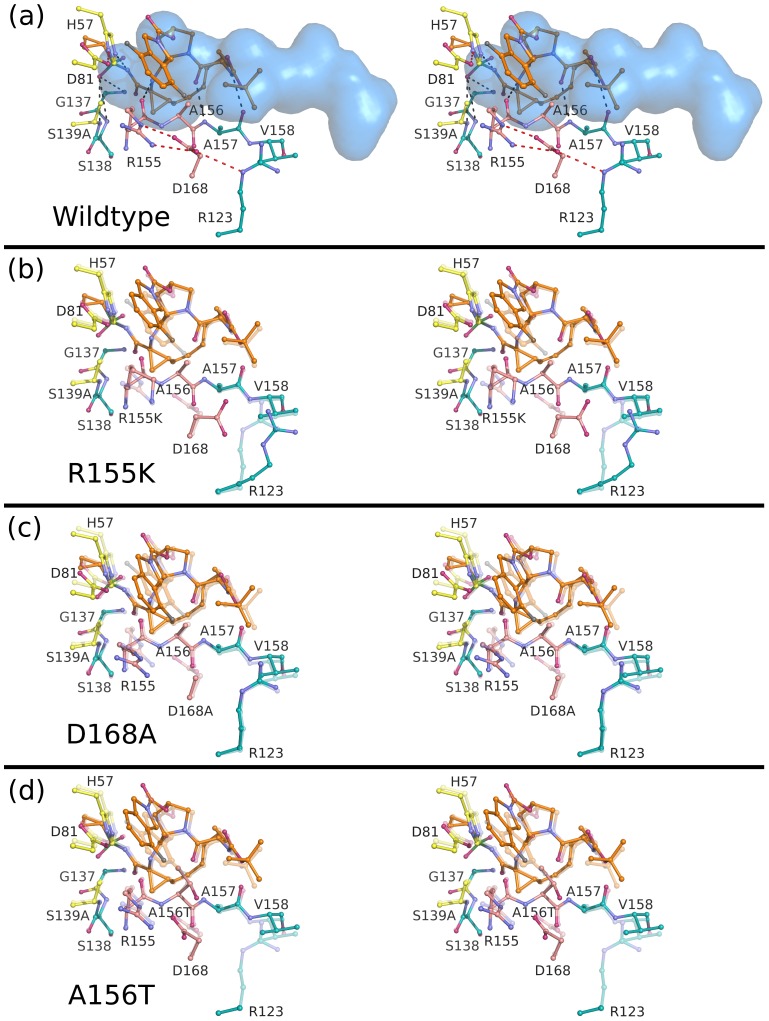
Stereo view of the danoprevir complexes. (A) Danoprevir bound to the wild-type protease with the substrate envelope in blue. Intra- and inter-molecular hydrogen bond interactions are marked as red and grey dashed lines. Danoprevir is also shown bound to the drug-resistant variants (B) R155K, (C) D168A and (D) A156T with the transparent coordinates representing the wild-type structure to better highlight the molecular changes of each mutation. In all cases, catalytic residues are depicted in yellow, the P2 subsite in pink, and the drug molecules in orange.

**Figure 5 ppat-1002832-g005:**
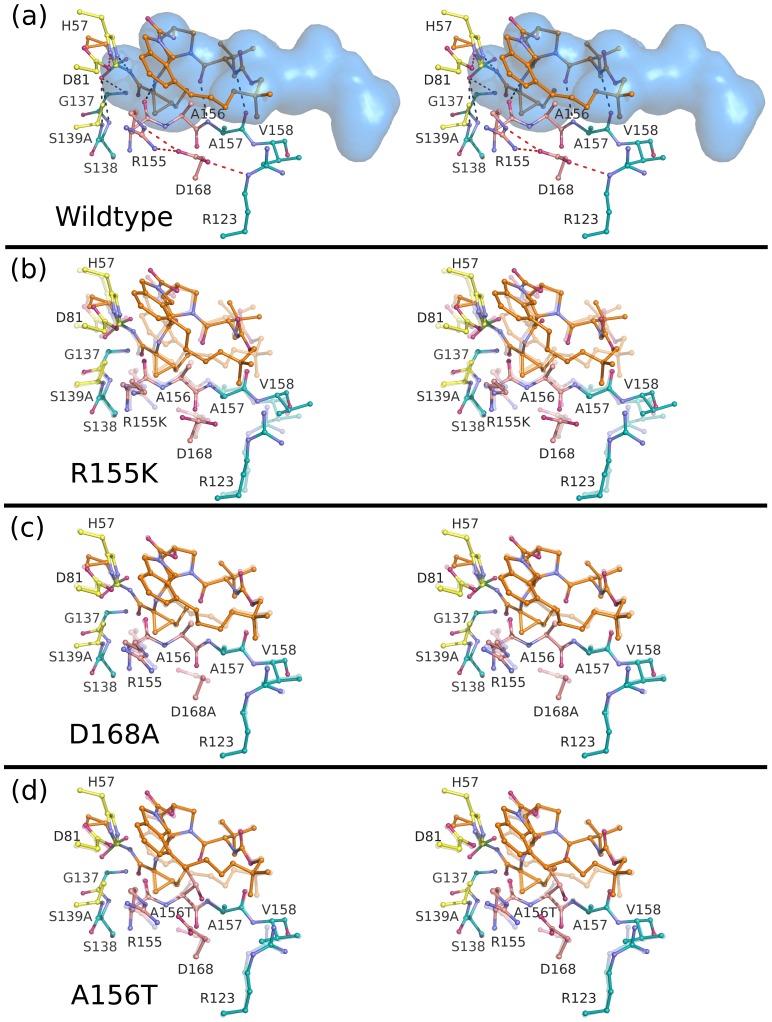
Stereo view of the vaniprevir complexes. (A) Vaniprevir bound to the wild-type protease with the substrate envelope in blue. Intra- and inter-molecular hydrogen bond interactions are marked as red and grey dashed lines. Vaniprevir is shown bound to the drug-resistant variants (B) R155K, (C) D168A and (D) A156T with the transparent coordinates representing the wild-type structure to better highlight the molecular changes of each mutation. In all cases, catalytic residues are depicted in yellow, the P2 subsite in pink, and the drug molecules in orange.

**Figure 6:**
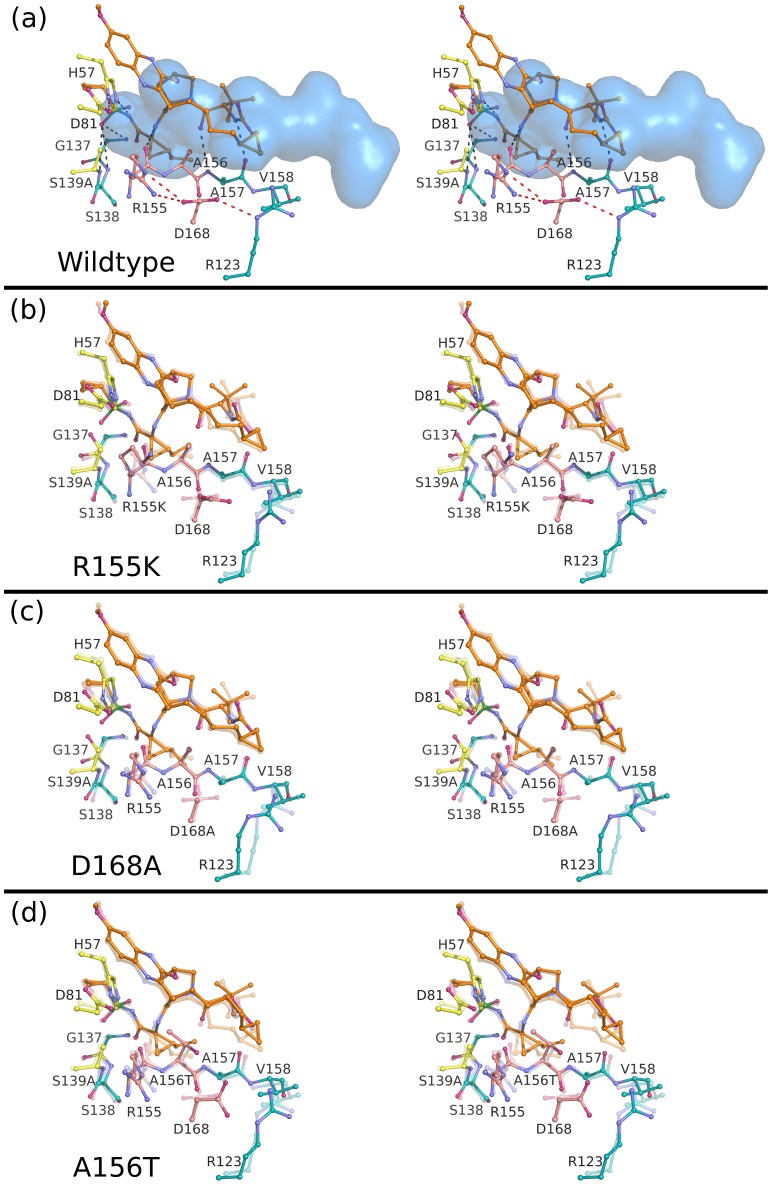
Stereo view of the MK-5172 complexes. (A) MK-5172 bound to the wild-type protease with the substrate envelope in blue. Intra- and inter-molecular hydrogen bond interactions are marked as red and grey dashed lines. MK-5172 is shown bound to the drug-resistant variants (B) R155K, (C) D168A and (D) A156T with the transparent coordinates representing the wild-type structure to better highlight the molecular changes of each mutation. In all cases, catalytic residues are depicted in yellow, the P2 subsite in pink, and the drug molecules in orange.

In wild-type complexes involving macrocyclic inhibitors, R155 adopted a conformation distinct from those observed in telaprevir and substrate complexes to allow binding of the extended P2 moieties in the S2 subsite. This R155 conformation is stabilized by hydrogen bond interactions involving D168 and D80. The conformation has also been observed for protease in complex with TMC435 and danoprevir, where large P2 moieties of inhibitors are positioned over the guanidine side chain, making extensive cation-π stacking interactions [43], [45]. Vaniprevir, with the P2 isoindoline moiety, bound in a conformation similar to danoprevir, making favorable cation-π stacking interactions with R155, despite the P2–P4 macrocycle. In contrast, MK5172 adopted a novel conformation with the ether-linked P2 quinoxaline moiety not interacting extensively with R155 and D168, but stacking instead against H57 and D81 of the catalytic triad (Figure 2). Thus, the P2 moieties of these three macrocycles pack in a variety of conformations around the active site.

To characterize binding patterns of the drugs relative to natural substrates, the wild-type drug complexes were analyzed with respect to the substrate envelope, the consensus binding volume of the substrates [43] (Figures 3A–[Fig ppat-1002832-g004] [Fig ppat-1002832-g005] 6A). Inhibitors are generally more vulnerable to resistance where they protrude beyond the substrate envelope and contact residues less essential for substrate recognition and turnover. All four drugs protruded from the substrate envelope in the protease S2 subsite near residues R155, A156 and D168, which individually mutate to confer multi-drug resistance [37], [38], [44]. Telaprevir, with the small P2 cyclopentylproline moiety, made fewer van der Waals contacts with R155, A156 and D168 relative to danoprevir and vaniprevir, which contain the carbamate-linked P2 isoindoline moieties that protruded from the substrate envelope and made extensive van der Waals contacts with these residues (Figures 3A–[Fig ppat-1002832-g004] 5A). Danoprevir's isoindoline moiety bound in two conformations in the wild-type complex, but adopted a single conformation in mutant complexes. Notably MK-5172, with an ether-linked P2 quinoxaline moiety, while protruding from the substrate envelope, stacked against the catalytic triad, avoiding direct van der Waals contact with R155 and D168 (Figure 6A). Thus, although each of these drugs protruded from the substrate envelope at the S2 subsite, each formed unique interactions with R155, A156 and D168. Mutations at these residues therefore differentially affected drug binding and potency, resulting in a distinct resistance profile for each inhibitor.

### Telaprevir resistance

**Figure 7:**
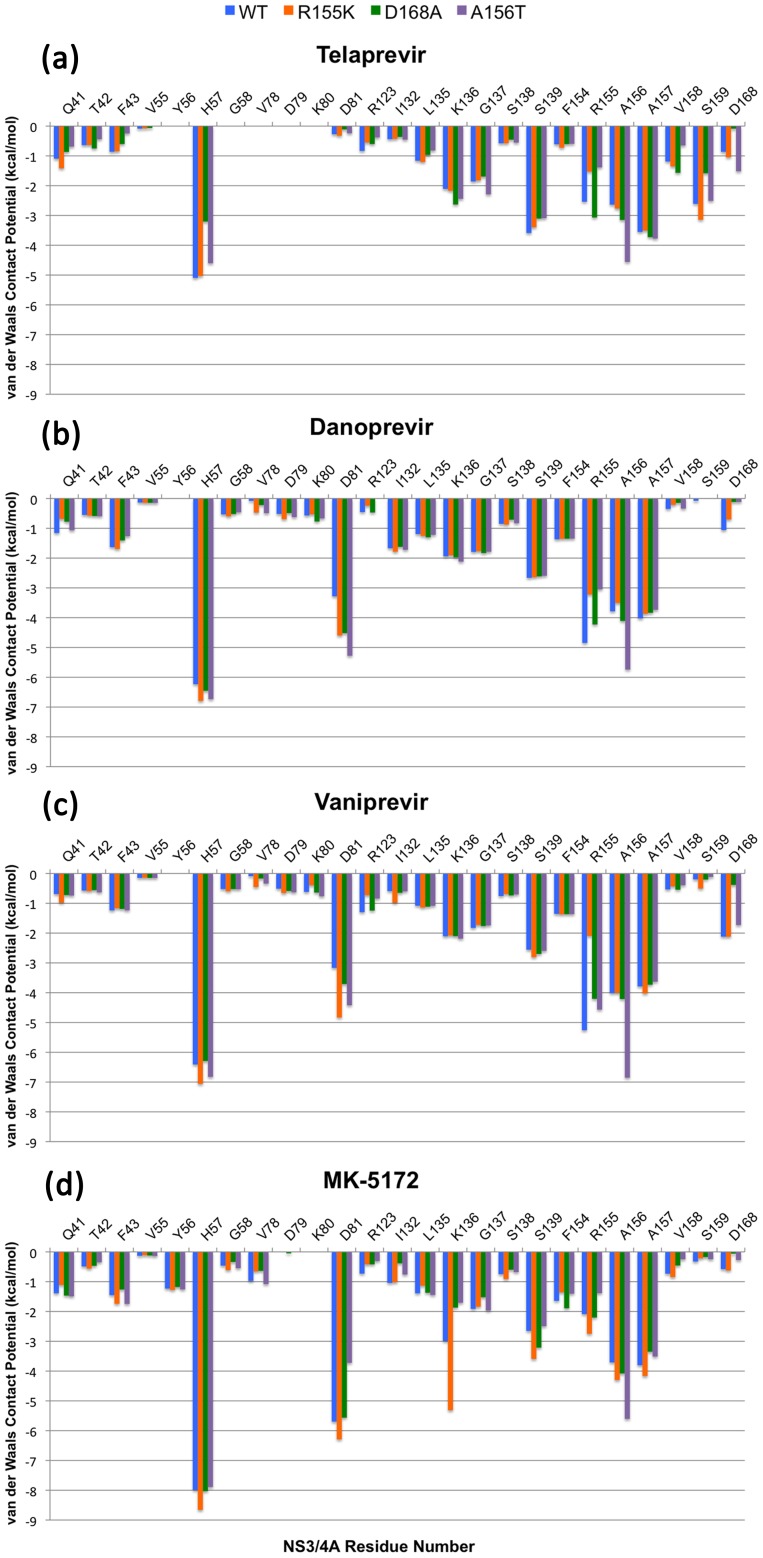
Drug interactions with wild-type and mutant complexes by residue. The Van der Waals contact energy indexes for the wild-type protease and mutant variants R155K, D168A and A156T are shown by protease residue for (A) telaprevir, (B) danoprevir, (C) vaniprevir and (D) MK-5172.

Telaprevir lost potency against R155K compared to the wild-type protease, although the crystal structures of both complexes were very similar maintaining the covalent bond between the ketoamide moiety and the catalytic serine (Figure 3B). R155K, however, lost interactions with D168, thereby disrupting the electrostatic network spanning R123, D168, R155 and D81, which is important for telaprevir binding. These rearrangements modulated the charge landscape along the protease surface, disrupting interactions with the adjacent P2 cyclopentylproline and P4 cyclohexylalanine moieties of telaprevir, consistent with previous modeling studies [48]. Interestingly, telaprevir showed better potency against the D168A variant than the wild-type; the crystal structure revealed that the P2 moiety bent considerably and packed closer against the D168A variant. The inhibitor shifted by approximately 0.5 Å relative the position in the wild-type complex, resulting in increased interactions with both R155 and A156 (Figures 3C, 7A). However, the A156T mutation resulted in a steric clash with telaprevir's P2 moiety, causing the inhibitor to shift significantly; the inhibitor P2 moiety moved away from R155, losing van der Waals interactions with the protease (Figures 3D, 7A). Notably, in the A156T-telaprevir complex the covalent bond between the ketoamide warhead and the catalytic serine was extended to greater than 2 Å, suggesting a reduced capacity for covalent modification, consistent with the large loss in potency against A156T (Table 1). Thus, while telaprevir's flexibility allows adaptation to D168A, it cannot accommodate the disruption by R155K or A156T. The relatively weak binding affinity of telaprevir to wild-type protease results in a potentially narrow range by which resistant mutations can be tolerated.

### Danoprevir and vaniprevir resistance

For both danoprevir and vaniprevir, the R155K mutation disrupted the favorable cation-π stacking interactions with the P2 isoindoline moieties (Figure 4B), causing significant reductions in drug potencies (Table 1). The D168A mutation also disrupted stacking of the P2 moieties with R155, by disrupting the electrostatic network and therefore the position of R155 for optimal cation-π stacking. In danoprevir, the P2 isoindoline moiety shifted in response to R155K and D168A mutations, making extensive interactions with the catalytic D81 (Figure 7B). For vaniprevir, the rigidity of the P2–P4 macrocycle prevented similar compensatory changes (Figure 5C). Thus, D168A caused losses in danoprevir and vaniprevir potency by disrupting cation-π stacking. However, the flexibility of the P2 moiety of danoprevir compensates somewhat for this loss, explaining danoprevir's greater potency against the D168A variant relative to vaniprevir (Table 1).

The A156T mutation sterically impinges on the binding of danoprevir and vaniprevir. In both complexes with A156T, the P2 moieties shifted toward the catalytic triad and lost cation-π stacking interactions with R155. However, the flexibility of the P2 moiety of danoprevir permitted a larger shift, which allowed for more compensatory packing against the A156T variant protease surface (Figure 4D). In contrast, the P2–P4 macrocycle of vaniprevir restrained the P2 moiety and inhibitor's ability to accommodate this steric burden, more strongly compromising the activity of vaniprevir. Thus, the flexible P2 moiety of danoprevir allowed it to retain significant potency against A156T variants compared to vaniprevir.

### MK-5172 resistance

Unlike in the danoprevir and vaniprevir complexes with wild type, in the MK-5172-wild-type complex the P2 quinoxaline moiety did not stack on R155 and interacted less with D168 and the electrostatic network involving these residues. Thus, the single-site mutations R155K and D168A only caused very subtle changes in the MK-5172 binding conformations (Figures 6B and 6C). This subtle effect is reflected in the small loss of potency against the R155K variant (Table 1); however, MK-5172 exhibited 100-fold lower potency against the D168A variant, likely due to less extensive interactions with D81 and K136 relative to wild-type and R155K (Figure 7). A156T, the worst of the resistance mutations for MK-5172A, sterically clashed with the P2–P4 macrocycle and caused a large shift in the binding position away from the catalytic triad relative to its wild-type structure (Figure 6D). This altered binding of MK-5172 resulted in fewer van der Waals contacts with D81 and R155, and is likely responsible for 1000-fold lower potency against the A156T variant. Overall, analysis of the four crystal structures explains MK-5172's significantly retained potency against R155K and D168A as well as its loss of potency against the A156T variant due to the rigidity of the macrocycle (Table 1).

## Discussion

Despite the exciting therapeutic success of NS3/4A protease inhibitors, their long-term effectiveness is challenged by drug resistance. In this study we explain the molecular basis of this drug resistance against four NS3/4A protease inhibitors, telaprevir, danoprevir, vaniprevir and MK-5172, representing the major chemical classes of these inhibitors. Our detailed analysis of 16 high-resolution crystal structures explains the loss of inhibitor potency in the face of resistance mutations. This research supports our substrate envelope model, which stipulates that inhibitors are vulnerable to resistance where they contact protease residues beyond the substrate-binding region and therefore are not essential for substrate binding [43]. These sites can mutate with minimal effect on protease function and viral fitness. Indeed, most resistance mutations occur in regions where drugs protrude from the substrate envelope, as these changes selectively disrupt drug binding with minimal effect on substrate proteolysis.

The most potent of the NS3/4A protease inhibitors is MK-5172. We report here, for the first time, a novel binding conformation for MK-5172 in which the P2 quinoxaline moiety binds far from the S2 subsite and instead stacks against the catalytic residues H57 and D81. Unlike other inhibitors, MK5172 does not directly interact with R155 and D168, which mutate to confer multi-drug resistance. This unique binding mode of MK-5172 explains its significantly greater potency against R155K and D168A variants compared to other inhibitors. MK-5172 has a unique barrier to resistance, as neither catalytic residue (H57 or D81) can tolerate mutation. This binding conformation of MK-5172, combined with its picomolar binding affinity [27] (Table 2), will likely allow it to retain potency against a broad array of resistant viral variants and genotypes.

We define the structural basis for differential drug activities against the resistant variants R155K, D168A, and A156T for four major chemical classes of NS3/4A protease inhibitors. Telaprevir has reduced potency against R155K due to loss of van der Waals contacts but exhibits better potency against D168A as it allows tighter packing in the S2 subsite. R155K and D168A mutations confer danoprevir and vaniprevir resistance by disrupting favorable cation-π stacking interactions with R155. Interestingly, while both drugs lose considerable potency against R155K, danoprevir retains higher activity against D168A. This difference is likely due to the flexible P2 isoindoline moiety of danoprevir, which lacks P2–P4 cyclization and repacks against the D168A variant. Similarly, vaniprevir and MK-5172 exhibit significantly lower potency against the A156T variant due to direct steric clashes, while danoprevir partially accommodates this steric burden by repacking against the mutated surface. Thus, the flexibility of danoprevir's P2 isoindoline moiety allows it to retain activity against two of the three major drug-resistant variants. Structural analysis of the 16 protease-inhibitor complexes defines the role of all three major drug-resistance mutations.

Our results also provide predictions of drug activities against other HCV genotypes and resistant strains. Interestingly, NS3/4A residues around the protease active site, including R155, A156, and D168 are highly conserved except genotype 3 viruses which contain the residues Q168 and T123, instead of D168 and R123 found in other genotypes (Figure S2). We predict that the terminal amide group of Q168 will be unable to stabilize R155 for stacking against the P2 moieties of danoprevir and vaniprevir, but may interact with T123 instead. Thus, we expect that danoprevir and vaniprevir will exhibit reduced potencies against genotype 3 viruses, while MK-5172 will remain fully active. Indeed, danoprevir and vaniprevir were recently shown to have reduced efficacy against genotype 3 viruses [49]. For genotype 1 strains, our results indicate that MK-5172 is highly active against R155K and D168A variants, while danoprevir is highly active against the A156T variant and to a lesser extent against the D168A variant. Thus, as new inhibitors are developed and HCV resistance testing becomes more available, our findings can help guide anti-HCV treatment regimens for individual patients.

Overall our findings correlate with resistance profiles observed in clinical isolates. Most protease inhibitors select for R155K variants in genotype 1a patients as only one nucleotide change is required [29], [30], [32], [37]–[42]. Genotype 1b patients presumably have higher barriers to R155K resistance, requiring two nucleotide substitutions; thus, mutations at A156 and D168 are more readily observed in response to protease inhibitor treatment. The resistance at R155K occurs due to reduced interactions in the S2 subsite. Telaprevir and other linear ketoamide drugs select for A156T variants [39]–[41] by direct steric clashes, while linear (BI 201335) and macrocyclic drugs (danoprevir, vaniprevir, TMC435) with large P2 moieties select for D168A variants [29], [30], [32], [42] by disrupting favorable stacking interactions with R155. These data also support the converse observation that D168A variants are uncommon in patients treated with telaprevir as the drug can pack tighter in the S2 subsite. Likewise, A156T variants are uncommon in patients treated with macrocyclic drugs containing flexible P2 moieties due to drug repacking against the mutated protease surface [29], [30], [32], [42]. However, drugs such as vaniprevir and MK-5172 containing P2–P4 cyclization likely select for the A156T variant due to the rigidity of their P2 moieties. Whether A156T variants will be found in clinical isolates, however, depends on additional viral factors, such as relative differences in viral fitness between A156T variants and other competing viral variants. Our data thus provide a unique resource for preemptively predicting resistance and choosing the most appropriate protease inhibitor to treat HCV depending on the resistance profile of a particular patient viral population. Whether or not specific mutations arise in clinical isolates is ultimately determined by the complex interplay between drug potency, viral fitness, and genetic barriers to resistance. Thus, depending on the initial viral species altered pathways to resistance will exist.

The crystal structures of these NS3/4A inhibitors also provide a key resource to guide future strategies in drug design. The high potency of MK-5172, for example, derives from interactions with the essential catalytic triad residues, which cannot mutate without severely disrupting viral fitness. In addition, flexible P2 drug moieties – lacking P2–P4 macrocycles – mitigate losses in potency to the A156T and D168A mutations. Similar chemical features can be incorporated in future drugs to potentially evade resistance. Specifically, novel protease inhibitors that incorporate flexible P2 moieties, such as quinoxaline or similar groups, could exploit interactions with the essential catalytic residues and concurrently minimize contact with the P2 subsite, thereby reducing their sensitivities to mutations at R155, D168 and A156T. Thus, our findings suggest strategies for developing protease inhibitors that retain activity against a wider spectrum of drug-resistant HCV variants.

## Materials and Methods

### Inhibitor synthesis

Danoprevir, vaniprevir and MK-5172 were synthesized in house following reported methods; danoprevir was prepared using our convergent reaction sequence as described [43]; vaniprevir and MK-5172 were prepared following the synthetic methods reported by McCauley *et al*. [50] and Harper *et al*. [27], respectively, with minor modifications. Telaprevir was purchased from A ChemTek, Inc. (Worcester, MA).

### Mutagenesis and gene information

The HCV genotype 1a NS3/4A protease gene described in a Bristol-Meyers Squibb patent [51] was synthesized by GenScript and cloned into the pET28a expression vector (Novagen). This highly soluble single-chain construct of the genotype 1a NS3/4A protease domain contains a fragment of the cofactor NS4A covalently linked at the N-terminus [51]. A similar protease construct exhibited catalytic activity comparable to that of the authentic full-length protein [52]. All protease variants were generated using the QuikChange Site-Directed Mutagenesis Kit from Stratagene. The codon-optimized genotype 1a helicase sequence (H77c) was cloned downstream to the protease gene to generate the full-length protease construct. Geneious [53] was used to generate the sequence alignment of the NS3/4A protease domain from HCV genotypes 1–6.

### Drug susceptibility assays

Single mutations (R155K, D168A, or A156T) were introduced into the NS3 region of genotype 1a HCV Con1 luciferase reporter replicon using the mega-primer method of mutagenesis [54]. Replicon RNA of each protease variant was introduced into Huh7 cells by electroporation. Replication was then assessed in the presence of increasing concentrations of protease inhibitors (telaprevir, danoprevir, vaniprevir or MK-5172) by measuring luciferase activity (relative light units) 96 hours after electroporation. The drug concentrations required to inhibit replicon replication by 50% (IC_50_) were calculated directly from the drug inhibition curves.

### Enzyme inhibition assays

For enzyme inhibition experiments, 5 nM of the genotype 1a HCV NS3/4A protease domain was incubated with increasing drug concentrations for 15 min (90 min for telaprevir) in 50 mM Tris assay buffer (5% glycerol, 5 mM TCEP, 6 mM LDAO and 4% DMSO, pH 7.5). Proteolysis reactions were initiated by adding 100 nM HCV NS3/4A substrate [Ac-DE-Dap(QXL520)-EE-Abu-ψ-[COO]AS-C(5-FAMsp)-NH2] (AnaSpec) and monitored using the EnVision plate reader (Perkin Elmer) at excitation and emission wavelengths of 485 nm and 530 nm, respectively. The initial cleavage velocities were determined from sections of the progress curves corresponding to less than 15% substrate cleavage. Apparent inhibition constants (*K*_i_) were obtained by nonlinear regression fitting to the Morrison equation of initial velocity versus inhibitor concentration using Prism 5 (GraphPad Software). Data for each drug were generated in triplicate and processed independently to calculate the average inhibition constant and standard deviation.

### Expression and purification of NS3/4A protease constructs

Protein expression and purification were carried out as described [51], [55]. Briefly, transformed BL21 (DE3) *E. coli* cells were grown at 37°C and induced at an optical density of 0.6 by adding 1 mM IPTG. Cells were harvested after 5 hours of expression, pelleted, and frozen at −80°C for storage. Cell pellets were thawed, resuspended in 5 mL/g of resuspension buffer (50 mM phosphate buffer, 500 mM NaCl, 10% glycerol, 2 mM β-ME, pH 7.5) and lysed with a cell disruptor. The soluble fraction was retained, applied to a nickel column (Qiagen), washed with resuspension buffer, and eluted with resuspension buffer supplemented with 200 mM imidazole. The eluent was dialyzed overnight (MWCO 10 kD) to remove the imidazole, and the His-tag was simultaneously removed with thrombin treatment. The nickel-purified protein was then flash-frozen and stored at −80°C.

### Crystallization of inhibitor complexes

The above-mentioned protein solution was thawed, concentrated to ∼3 mg/mL and loaded on a HiLoad Superdex75 16/60 column equilibrated with gel filtration buffer (25 mM MES, 500 mM NaCl, 10% glycerol, 30 µM zinc chloride, and 2 mM DTT, pH 6.5). The protease fractions were pooled and concentrated to 20–25 mg/mL with an Amicon Ultra-15 10 kD device (Millipore). The concentrated samples were incubated for 1 hour with 1–3 molar excess of inhibitor. Diffraction-quality crystals were obtained overnight by mixing equal volume of concentrated protein solution with precipitant solution (20–26% PEG-3350, 0.1 M sodium MES buffer, 4% ammonium sulfate, pH 6.5) in 24-well VDX hanging drop trays.

### Crystallization, data collection and structure solution

X-ray diffraction data were collected at Advanced Photon Source LS-CAT 21-ID-F, GM/CA-CAT 23-ID-D or with the in-house RAXIS IV X-ray system. Diffraction intensities were indexed, integrated and scaled using the program HKL2000 [56]. All structure solutions were generated using simple isomorphous molecular replacement with PHASER [57]. The B chain model of viral substrate product 4A4B (3M5M) [43] was used as the starting model for all structure solutions. Initial refinement was carried out in the absence of modeled ligand, which was subsequently built in during later stages of refinement. Subsequent crystallographic refinement was carried out within the CCP4 program suite, with iterative rounds of TLS and restrained refinement until convergence was achieved [58]. The protein crystals of the wild-type protease and drug-resistant variants R155K and D168A in complex with MK-5172 grew as pseudo-merohedral twins. Amplitude-based twinned refinement was carried out during restrained refinement for all pseudo-merohedral twins. The final structures were evaluated with MolProbity [59] prior to deposition in the Protein Data Bank. To limit the possibility of model bias throughout the refinement process, 5% of the data were reserved for the free R-value calculation [60]. Interactive model building and electron density viewing was carried out using the program COOT [61]. *F_obs_−F_calc_* ligand omit maps were generated with the ligand excluded from the phase calculation using the program PHENIX [62].

### Inhibitor complex analysis

Superpositions were performed in PyMOL [63] using the Cα atoms of the active site protease residues 137–139 and 154–160. The wild-type-danoprevir complex was used as the reference structure for each alignment. The NS3/4A viral substrate envelope was computed as described using the full-length NS3/4A structure (1CU1) [64] and product complexes 4A4B (3M5M), 4B5A (3M5N) and 5A5B (3M5O) [43].

### Van der Waals contact energy

Van der Waals contact energies between protease residues and peptide products were computed using a simplified Lennard-Jones potential as described [65]. Briefly, the Lennard-Jones potential (*V_r_*) was calculated for each protease-drug atom pair where *r*, ε and σ represent the interatomic distance, vdW well depth, and atomic diameter, respectively:
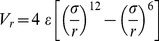

*V_r_* was computed for all possible protease-drug atom pairs within 5 Å, and potentials for non-bonded pairs separated by less than the distance at the minimum potential were equated to −ε.

## Supporting Information

Figure S1 **Ligand omit maps for protease-inhibitor complexes.** The NS3/4A wild-type protease (grey cartoon) is shown with inhibitors (orange sticks): (A) telaprevir, (B) danoprevir, (C) vaniprevir and (D) MK-5172. The electron density maps (blue) depict the *F_obs_* ***−*** *F_calc_* ligand omit maps contoured at 1σ.
(TIF)

Figure S2 **Sequence alignment of the NS3/4A protease domain for HCV genotypes 1–6.** Consensus sequence (1a M62321) of NS3/4A protease domain is shown in grey. Amino acid residues in disagreement are highlighted in color. Residues at positions 155 and 156 are conserved across genotypes; however, genotype 3 shows divergence from the consensus at amino acid 168.
(TIF)

Table S1 **Drug hydrogen bonds and vdW contacts with wild-type protease.**
(DOC)
