# Supplementary figures and images for: The Molecular Basis of Drug Resistance against Hepatitis C Virus NS3/4A Protease Inhibitors
Source: PLoS Pathog. 2012 Jul 26;8(7):e1002832. doi: 10.1371/journal.ppat.1002832 (PMC3406087; doi:10.1371/journal.ppat.1002832)

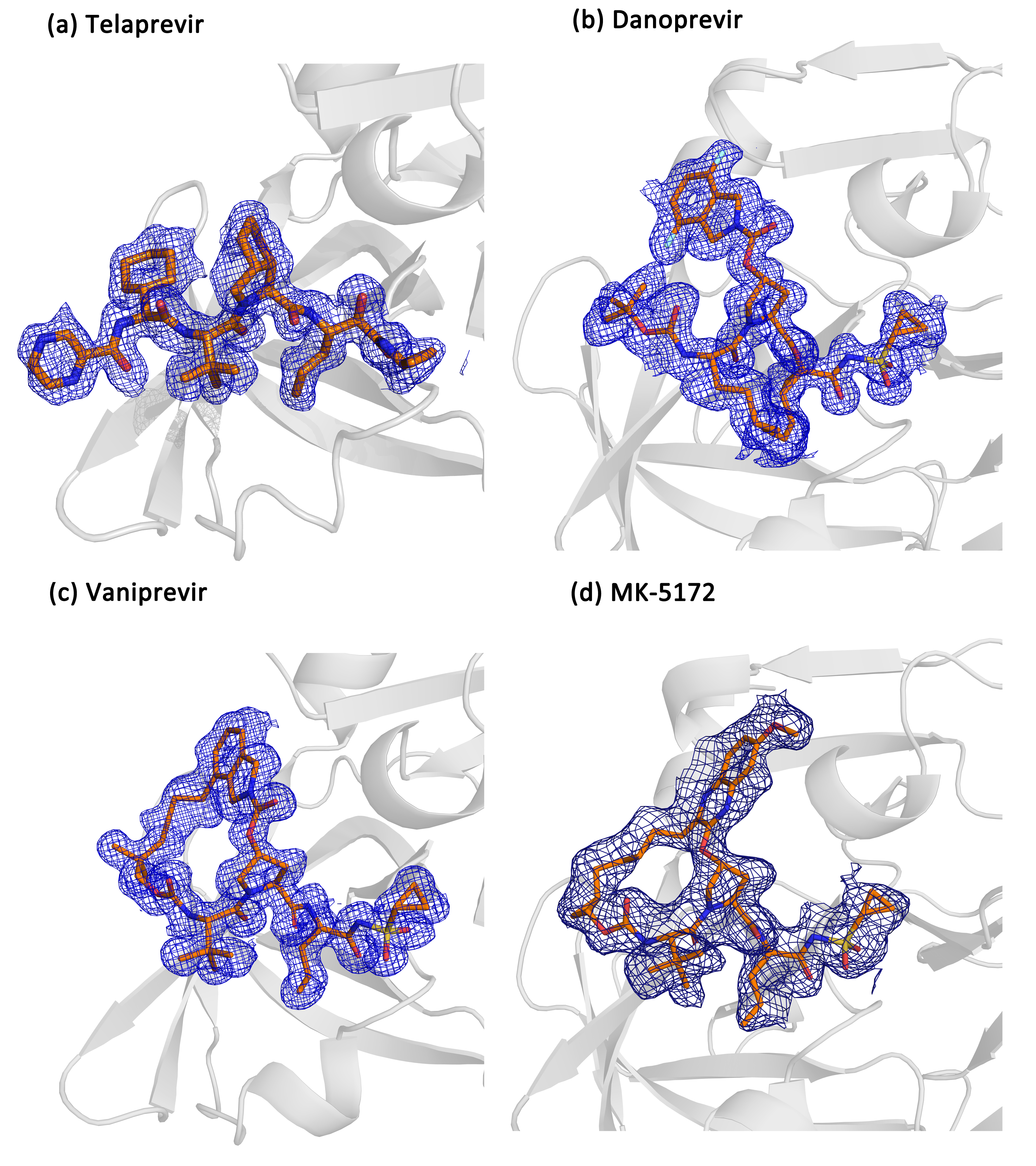

Supplement: Figure S1 — Ligand omit maps for protease-inhibitor complexes. The NS3/4A wild-type protease (grey cartoon) is shown with inhibitors (orange sticks): (A) telaprevir, (B) danoprevir, (C) vaniprevir and (D) MK-5172. The electron density maps (blue) depict the Fobs − Fcalc ligand omit maps contoured at 1σ. (TIF) [file ppat.1002832.s001.tif]

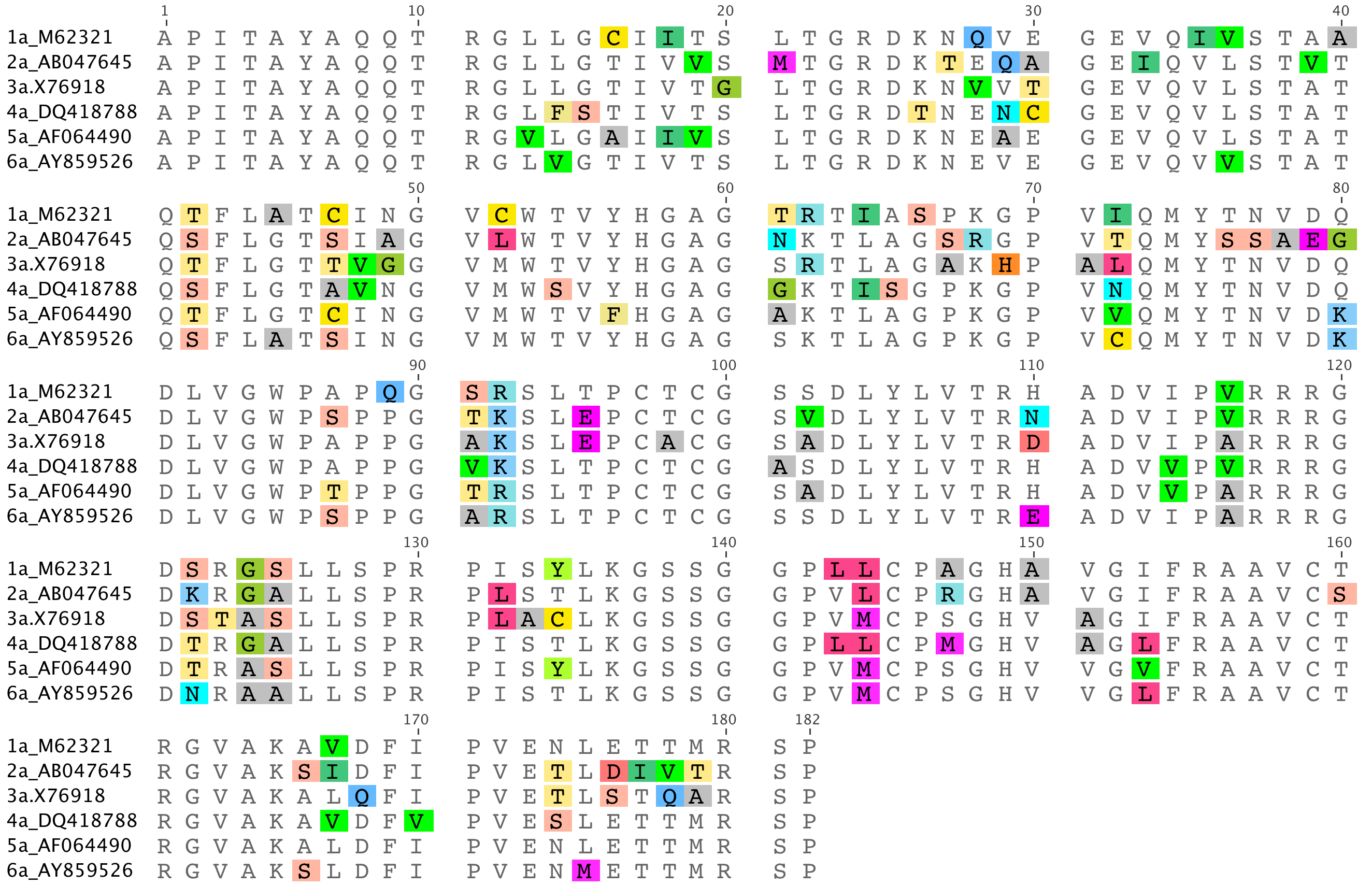

Supplement: Figure S2 — Sequence alignment of the NS3/4A protease domain for HCV genotypes 1–6. Consensus sequence (1a M62321) of NS3/4A protease domain is shown in grey. Amino acid residues in disagreement are highlighted in color. Residues at positions 155 and 156 are conserved across genotypes; however, genotype 3 shows divergence from the consensus at amino acid 168. (TIF) [file ppat.1002832.s002.tif]
