# Supplementary material for: The Molecular Basis of Drug Resistance against Hepatitis C Virus NS3/4A Protease Inhibitors
Source: PLoS Pathog. 2012 Jul 26;8(7):e1002832. doi: 10.1371/journal.ppat.1002832 (PMC3406087; doi:10.1371/journal.ppat.1002832)
Supplement: Table S1 — Drug hydrogen bonds and vdW contacts with wild-type protease. (DOC) [file ppat.1002832.s003.doc]

| **Table S1**. Drug hydrogen bonds and vdW contacts with wild-type protease. | | | | | | | | |
| --- | --- | --- | --- | --- | --- | --- | --- | --- |
| ***Moiety*** | ***Telaprevir*** | | ***Danoprevir*** | | ***Vaniprevir*** | | ***MK-5172*** | |
|  | ***Hbonds*** | ***VdWs*** | ***Hbonds*** | ***VdWs*** | ***Hbonds*** | ***VdWs*** | ***Hbonds*** | ***VdWs*** |
| **P1'** | G137  S138  S139 | Q41  T42  F43  K136 | G137  S138  S139A | Q41  T42  F43  K136 | G137  S138  S139A | Q41  T42  F43  K136 | G137  S138  S139A | Q41  T42  F43  K136 |
| **P1** | H57  R155 | F154 | H57  R155 | F154 | H57  R155 | F154 | H57  R155 | F154 |
| **P2** |  | R155  A156  D168 |  | R155  A156  D168  D79  K80 |  | R155  A156  D168  D79  K80 |  | Y56  H57  D81  R155  A156 |
| **P3** | A157^a^ | I132  L135  A157 | A157^a^ | I132  L135  A157 | A157^a^ | I132  L135  A157 | A157^a^ | I132  L135  A157 |
| **P4** |  | R123  A156  V158  D168 |  | A156  D168 |  | R123  D168 |  | R123  V158 |
| **P5** | S159 | S159 | - | - | - | - | - | - |
| ^a^Contributes two hydrogen bonds | | | | | | | | |
